# Menopause knowledge, attitudes and experiences of women in Saudi Arabia: a qualitative study

**DOI:** 10.1186/s12905-024-03456-7

**Published:** 2024-11-25

**Authors:** Ghada AlSwayied, Rachael Frost, Fiona L. Hamilton

**Affiliations:** 1 UCL Research Department of Primary Care and Population Health, University College London, Upper 3rd Floor, Royal Free Campus, Rowland Hill Street, London, NW3 2PF UK; 2 Department of Community Health Sciences, King Saud University, Riyadh, Saudi Arabia; 3 Health and Social Care Department, Liverpool John Moores University, Liverpool, UK

**Keywords:** Women, Menopause, Menopausal transition, Perimenopause, Menopausal symptoms, Self-care, Culture, Saudi Arabia

## Abstract

**Background:**

Menopause can be seen as a complex phenomenon influenced by an individual’s cultural norms, belief systems, and lifestyle choices. In conservative societies such as Saudi Arabia, some women silently struggle with menopause due to cultural taboos and social stigma, making their experiences invisible and lowering their quality of life. The aim of this study was to explore in-depth the knowledge, attitudes, and experiences of middle-aged Saudi women with menopause.

**Methods:**

The study employed qualitative methods using semi-structured interviews with middle-aged women undergoing menopause in Saudi Arabia from February to May 2023. The interviews were conducted online in Arabic, recorded and transcribed verbatim. The data were analysed inductively using thematic analysis.

**Results:**

A total of twenty-nine women, aged 40–64 years, were interviewed. Three key themes were identified: mixed emotions towards menopause; experiencing biopsychosocial changes; and adapting to the transition. Overall, most participants had mixed perspectives on menopause. Negative aspects included feelings of uncertainty and the loss of fertility while positive aspects included a sense of relief from menstruation and the newfound freedom to engage in religious activities at any time. Notably, during the interviews, many participants reconsidered their initial negative views and voiced predominantly positive perspectives. Several women reported inconvenient menopausal symptoms, such as hot flashes, night sweats, disturbed sleep, and fatigue. Concerns about being perceived as less attractive led many to keep their symptoms private, and societal expectations played a significant role in influencing how women managed these symptoms and sought help. Many women opted to seek information discreetly online rather than seeking support from others. Self-care practices were favoured for managing menopause, with medical care being undervalued or at times deemed unsatisfactory.

**Conclusion:**

In Saudi Arabia, menopause is generally seen as a natural phase of life. Many women appreciate the positive aspects it brings, such as relief from menstrual pain and an enhanced ability to participate in religious practices. However, they also encounter challenges, including hot flashes and concerns about perceived decreases in attractiveness. A notable trend is the preference for self-care strategies over medical interventions or hormone replacement therapy (HRT). There is a need to raise awareness about menopausal symptoms to reduce negative perceptions and experiences and to develop health promotion and educational interventions to support and empower women during this transition. Future research with healthcare professionals would extend these findings.

**Supplementary Information:**

The online version contains supplementary material available at 10.1186/s12905-024-03456-7.

## Introduction

Menopause is the cessation of menstruation and is usually confirmed after twelve consecutive months of amenorrhea [1]. The WHO predicts that by 2050, there will be one billion perimenopausal or postmenopausal women [2].

Saudi Arabia, the largest high-income country in the Arabian Peninsula, has a population exceeding 36 million, with women accounting for 42% of the population [3]. By 2050, the population is projected to reach 40.4 million, with 25% aged 60 years or older [4]. Observational studies conducted in Saudi Arabia revealed that the average age of natural menopause is approximately 48.98 years, with a median age of 50 years [5, 6]. Consequently, it is estimated that approximately one-third of a woman’s lifespan in Saudi Arabia will be postmenopausal.

The menopausal transition, also known as perimenopause, is a unique process for each woman and can last between two and eight years before menopause [7]. A decrease in oestrogen levels and fluctuations in menstrual periods leads to physical and psychological symptoms [7]. Commonly reported vasomotor symptoms, such as hot flashes and night sweats, affect up to 80% of menopausal women [8]. Other symptoms may include fatigue, difficulty concentrating, insomnia, weight gain, mood swings, decreased sexual function, vaginal dryness, urinary incontinence, anxiety, irritability, and depression [9–11]. Studies have shown that the prevalence of menopausal symptoms among Saudi women is similar to that reported in studies of both Asian and Western women [12]. Additionally, several quantitative studies have indicated that Saudi women may perceive the perimenopause transition as challenging, resulting in a moderately low quality of life [13, 14].

The concept of menopause, viewed through a social anthropological lens, can be seen as a multifaceted phenomenon shaped by an individual’s cultural norms, belief systems, and lifestyle choices [15, 16]. Menopause is deeply embedded within the cultural and religious framework of patriarchy [17]. Existing studies have shown that sociocultural factors can affect women’s attitudes toward, perceptions of, and experiences with menopause. Notably, the literature from Islamic Asian countries, such as Pakistan [18], Iran [19], and Malaysia [20], suggest that women often underestimate the significance of menopause and lack comprehensive knowledge to address menopausal symptoms effectively. Owing to cultural taboos and social stigma, some women silently grapple with menopause, making their experiences invisible [21]. Therefore, it is essential to explore knowledge and attitudes towards menopause while considering the impact of culture on this journey [22].

In Arabic, the term used to describe menopause is “Sen al Yaas”, which means “the age of despair or hopelessness” carrying a negative connotation, contributing to the taboo surrounding this topic. Nevertheless, there have been some media efforts to replace the negative term with a more positive one, “the age of wisdom” [23].

In recent years, several observational studies have examined menopause in different regions of Saudi Arabia, focusing on the frequency and severity of menopausal symptoms, quality of life, and influence of sociodemographic factors, mainly using cross-sectional designs and self-reported questionnaires [12, 14, 24–29]. There is limited exploration of the lived experience of menopause and sociocultural aspects, including the barriers and facilitators to seeking information and support for menopause.

This qualitative study therefore explores the attitudes and experiences of middle-aged Saudi women regarding menopause. It aims to understand women’s comprehension of menopause, their symptom management preferences, and challenges in seeking support and information. By using qualitative research methods, this study aims to fill this research gap and inform the development of tailored menopause support strategies and programs for policymakers in the region.

## Methods

### Study design

This study employed a qualitative research design involving 29 middle-aged women in Saudi Arabia. Qualitative research methods are suitable for exploring perceptions, attitudes, and experiences to gain a thorough understanding of sociocultural contexts [30]. The research was underpinned by a social constructivism theoretical framework [31]. The consolidated criteria for reporting the 32-item checklist of qualitative studies guided the reporting of this study [32].

One-on-one, semi-structured online interviews were conducted to gain in-depth insights into the experiences of menopause among Saudi women. This approach was chosen over focus group discussions after input from public and patient involvement (PPI) contributors to allow for open and comprehensive discussions on sensitive health topics such as women’s attitudes and experiences related to intimate and sexual changes during menopause, in addition to capturing diverse perspectives on the topic [33]. After careful deliberation and consultation with PPI, the decision was made to opt for online interviews as opposed to in-person ones. This choice was prompted by the impracticality of holding interviews on the topic of menopause in public settings and the added benefit of allowing women to participate from the comfort of their homes, addressing transportation issues. During the interviews, the participants were encouraged to freely express their views and experiences, facilitating a natural flow of conversation.

### Patient and public involvement

This research was guided by two PPI members, both middle-aged Saudi women undergoing menopause. Translated versions of the research materials were reviewed by the PPI members, who then provided feedback in both written and verbal forms during an informal discussion session. This feedback was incorporated into a draft of the participant information sheet, informed consent, and study poster. A subsequent session was held to review the topic guide in terms of the relevance of the covered topics and the clarity and wording of the questions. The suggested changes to the topic guide by the PPI members were carefully considered in the final draft. A concise summary in Arabic of the initial themes identified was shared with the PPI members to gather their insights into the findings.

### Participants

We conducted interviews with Saudi women aged between 40 and 64 years including those in the pre-menopausal, perimenopausal, and post-menopausal stages, to capture a comprehensive understanding of their experiences throughout the menopause transition. We included women from the community with and without health conditions, including those with multiple chronic diseases. Women were eligible to participate if they had gone through natural menopause, defined as the natural cessation of menstrual periods, or sudden menopause due to cancer treatments or surgeries, such as breast and cervical cancer survivors. Women were eligible regardless of the use of hormonal replacement therapy (HRT). The participants needed internet access on the day of the interview to take part in the online interviews. However, owning a smartphone was not a criterion for participation in the study to minimise the digital divide.

### Sampling and recruitment

Women were purposively sampled on the basis of age range, marital status, educational background, employment status, and geographical location (region). We further theoretically sampled women on the basis of menopausal stage (premenopausal, perimenopausal and post-menopausal) to ensure our sample captured a wide variety of views and experiences.

Recruitment for the study spanned 16 weeks beginning in January 2023. Prospective participants were identified through various social media platforms, including WhatsApp, Twitter, and Instagram. Snowball recruitment was encouraged through the WhatsApp broadcast feature. Study posters were also distributed in supermarkets, pharmacies, and beauty salons in Riyadh, the capital city of Saudi Arabia, to help reach women who may have lower digital literacy and are less likely to be reached via social media recruitment.

Interested participants were directed to a research website where they could access detailed information prior to completing the electronic informed consent and demographic questionnaire. Out of the various recruitment channels used to reach potential participants, 86 women expressed their initial interest and successfully provided informed consent and completed a sociodemographic questionnaire in Microsoft Forms. Upon submission of the consent form, an automatic email was sent to every interested participant to book a slot for the interview on Calendly platform. Two weekly reminders were sent via emails and WhatsApp messages to those who did not confirm a slot for the interview despite submitting their consent forms and questionnaires. Recruitment continued until no further new themes were identified and meaning (information) saturation was reached after the 29th interview. This concept refers to achieving a deep understanding of the data, a point in the data collection and analysis process where no new information about the meaning of codes or themes emerges [34, 35].

### Data collection and topic guide

Data were collected from January 2023 to April 2023. All the interviews were conducted online in Arabic via Microsoft Teams by the primary investigator (GS) and lasted for 45–60 min. The participants were not required to have cameras on. With the participants’ permission, interviews were audio-recorded using Microsoft Teams, and verbal as well as written confirmation of their consent was obtained.

The topic guide structured the interview discussions around the women’s lived experiences with menopause, including questions about their personal perspectives on menopause, menopausal knowledge, the symptoms and changes they experienced, where they gained information and support, how they managed the symptoms, and the strategies they used to cope [see supplementary material 1: The Topic Guide]. Throughout the study, the topic guide was refined iteratively to ensure a comprehensive exploration of relevant areas of interest. This involved providing input from consultants and experts, as well as piloting the interview guide with the PPI members to make necessary adjustments on the basis of their feedback. The pilot interviews were not included in the analysis.

### Data analysis

The interviews transcribed verbatim in Arabic by the primary researcher (GS) to facilitate data analysis. The data were analysed inductively via reflexive thematic analysis as described previously [36], encompassing six stages: data familiarisation, coding, theme identification, revision and reviewing, naming and redefining themes, and finally, interpretation of common patterns across the dataset. Constant comparative analysis was conducted by exploring common themes in subsequent interviews until meaning saturation was achieved [34, 35]. Two comprehensive interviews were fully translated into English to obtain feedback from non-Arabic speaking coinvestigators (FH and RF). Relevant excerpts were also translated into English where needed, with a random sample back-translated into Arabic to check accuracy.

ATLAS.ti software was used to code the transcripts line by line, highlighting key words or phrases from the text. The codes were initially created by the lead researcher (GS) and then revised and refined through iterative discussions with experienced researchers (FH) and (RF) to ensure transparency and critical analysis. The codebook underwent multiple revisions and amendments until a consensus was reached. Afterward, the codes were organised into meaningful clusters to identify initial themes across the dataset. The preliminary thematic framework was continually refined throughout the data analysis and writing stages, incorporating input from all the authors. The diverse cultural perspectives provided by coinvestigators have made significant contributions to the analysis [37].

### Rigour of the study

To help build good rapport and increase the credibility of the data, the first author interviewed all the women in their native language (Arabic) and a summary of findings was presented to a group of participants for feedback on the identified themes. We also aimed for maximum variance in participants’ age range, education levels, employment, marital status, and geographical location. To ensure dependability and confirmability, the entire thematic analysis process was thoroughly reviewed by all three authors, and quotes were used to support themes. To facilitate transferability, a detailed description of the studied context, characteristics and experiences of the women involved is included.

### Reflexivity

The first author (GS) is from Saudi Arabia and has extensive context-specific knowledge about Saudi Arabian culture and the national healthcare system. The second and third authors (RF and FH) are senior researchers with a wealth of expertise in qualitative research and thematic analysis.

### Ethical considerations

This study received approval from the UCL ethics committee with reference number 23,817/001 in London, UK. Additionally, approval was obtained from the local ethics committee at King Saud University in Riyadh, Saudi Arabia (reference number: 22/0477/IRB). All participants were provided with both verbal and written information about the research and consented online via Microsoft Forms before taking part. The participants’ information was pseudonymized to ensure their confidentiality.

## Results

### Sociodemographic characteristics

Twenty-nine Saudi women between the ages of 40 and 64 years were interviewed (Table 1). All participants were Saudi citizens living in Saudi Arabia and identified Islam as their religion. The majority of women self-identified as *‘perimenopausal’* (*n* = 18), with five *‘post-menopausal’* women and six *‘premenopausal’* women. Twenty-four participants were married and lived with their spouses, four were divorced or widowed, and one was never married. Many women had higher education: 15 had bachelor’s degrees, seven had postgraduate qualifications, and seven had secondary education or lower. Thirteen were housewives, eight worked full-time, three worked part-time, and four had retired in the last two years.

**Table 1:** Demographic data of the participating women (*N* = 29)

Participants	Saudi women	*N* = 29
Age range	40– <45	7
	45– <50	5
	50– <55	6
	55– <60	7
	60–less than 65	4
Menopause stage	Pre-menopausal (not yet – borderline, regular menstruation in last 3 months, no menopausal symptoms)	6
	Perimenopausal (currently struggling with irregular menstruation and mild/severe menopausal symptoms)	18
	Post-menopausal (previously experienced menopause symptoms, ≥ 12 months without menstruation)	5
Menopause type	Natural menopause	23
	Sudden menopause (also known as induced menopause due to surgeries or medical treatments such as chemotherapy)	6
Marital status	Married	24
	Never married	1
	Divorced	2
	Widowed	2
Motherhood	Have children	22
	No children	7
Highest education qualification	Primary (Elementary school)	1
	Middle school	1
	Secondary (High school)	5
	Bachelor’s	15
	Master’s	5
	PhD	2
Employment status	Housewife	13
	Employed (full-time)	8
	Employed (part-time)	3
	Self-employed (freelancer work from home)	1
	Retired	4
Region of residence	Central	15
	Eastern	7
	Western	6

### Thematic findings

**Fig. 1:**
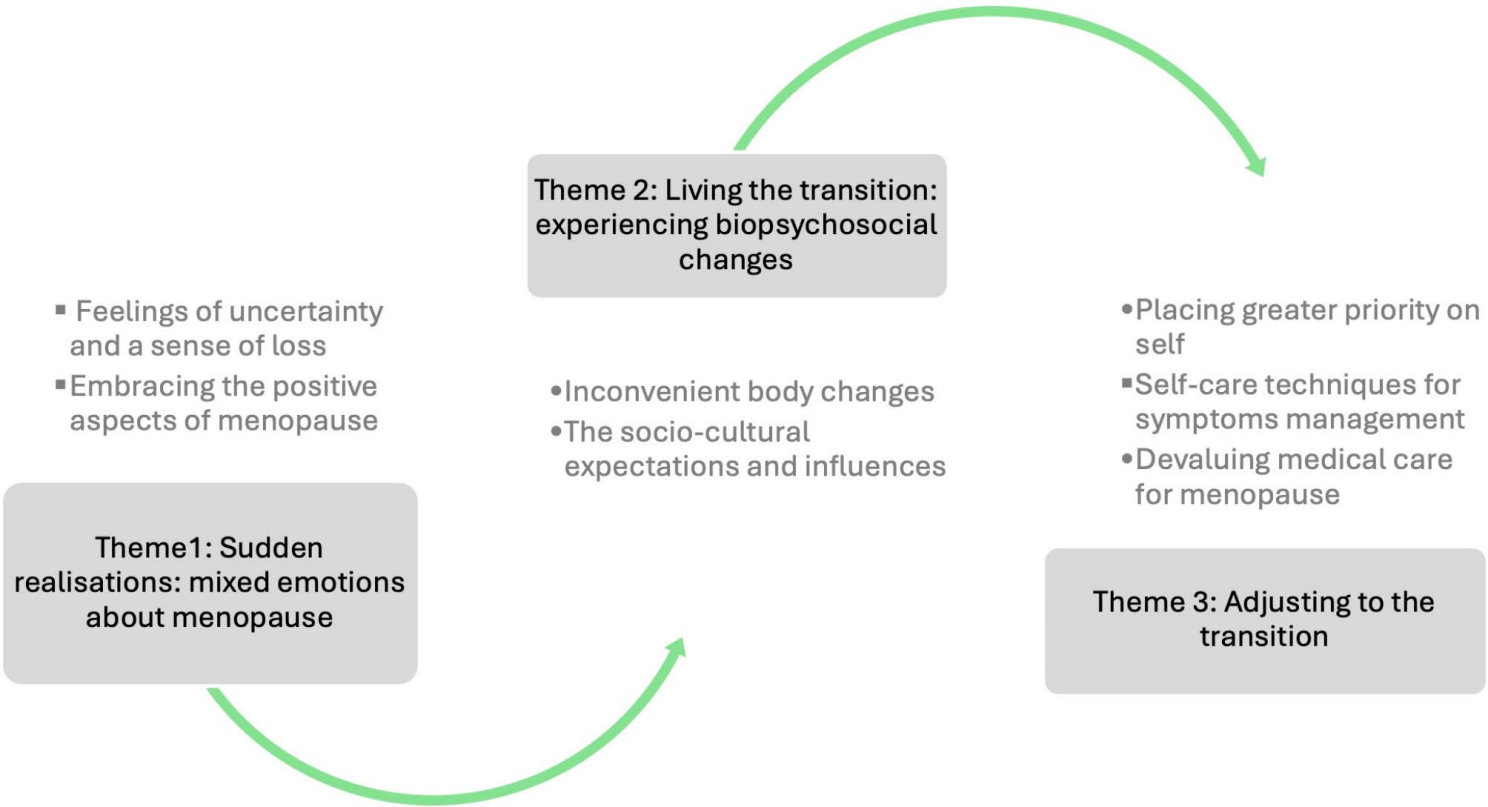
Thematic framework of menopause perceptions and experiences among middle-aged women in Saudi Arabia

Among the participants, three key themes were identified: conflicting emotions towards menopause, experiencing biopsychosocial changes, and adjusting to the transition process. A conceptual thematic framework, illustrated in Fig. 1, outlines the key themes that appeared to unfold throughout their menopausal journey. These themes are supported by subthemes and excerpts from the interviews with the participants.

#### Theme (1): Mixed emotions towards menopause

Most participants expressed having mixed perspectives on menopause, but predominantly emphasised the positive aspects of this stage of life and viewed it as a natural and normal phase.

##### Sudden realisations: feelings of uncertainty and a sense of loss

The transition to menopause often triggered negative emotions in many women, as they grapple with the sudden realisation of the process. Participants characterised the perimenopause as a time of uncertainty, leading to inner turmoil: *“In the beginning*, *the realisation that my period has stopped permanently left me with countless unanswered questions. Every woman goes through this battle* (معركة) *alone*” (P19, age range 50–54, bachelor’s, married, full-time job).

Most participants reported a lack of knowledge about menopause and were unprepared for the accompanying changes, leading to unnecessary worries. For example, some women misinterpreted common symptoms such as hot flashes and night sweats as more serious issues: *“When I started experiencing hot flashes*, *I became worried and scared about the link between body heat and cancer*, *as no one had spoken to me about it before.”* (P25, age range 55–59, Bachelor’s, married, housewife).

Most women acknowledged menopause as a natural and inevitable phase marking the end of the menstrual cycle and their reproductive phase. However, women who had not given birth displayed a greater likelihood of entering menopause with a negative outlook, with sustained emotions such as sadness and grief attributed to their diminishing fertility: *“It was not easy for me. I mean*, *at first*, *I felt like I had lost something”* (P18, age range 55–59, Bachelor’s, married, part-time job).

Similarly, the women who had undergone sudden menopause after cancer treatment perceived menopause as a significant hurdle due to the intensity of menopausal symptoms and the emotional challenge of coming to terms with the unnatural aspects of their menopausal journey, which had an additional impact alongside their illness. One woman commented, *“I am 42*, *and my period stopped after the first chemo session. It has been nine months since then*, *and to be honest*, *I am not sure how I feel about menopause. My illness made my emotions confusing and hard to process*, *so it is difficult for me to have a clear perspective on menopause itself”* (P1, aged 40–45, secondary education, married, housewife).

##### Embracing the positive aspects of menopause

During the interviews, many participants have challenged their negative perceptions of menopause with optimistic ones, expressing a predominantly positive outlook. For instance, one participant, who initially described menopause as a “*battle* (معركة),” also viewed it as an opportunity for personal growth and self-discovery, emphasising, “*At the same time*, *[…] I see it as a new chapter in a woman’s life*, *characterized by heightened wisdom and emotional growth*” (P19, aged 50–54, bachelor’s degree, married, full-time job). Similarly, another participant chose to refer to menopause as the “*Age of hope and wisdom*” (P10, aged 50–54, bachelor’s, married, retired) instead of the commonly used Arabic term ‘سن اليأس, Sen el Yaas,’ (“*Era of Despair*”).

Relatedly, many participants expressed finding comfort in their religious beliefs during this crucial phase. For some, their unwavering faith in Allah played a vital role in empowering them to navigate negative thoughts and embrace this new chapter in life with acceptance: “*I believe that my faith in Allah has helped me accept the change. I saw the cessation of menstruation as a divine blessing*” (P21, aged 55–59, PhD, married, full-time job).

Menopause was also regarded positively as a period of relief and relaxation when the discomfort and pain associated with menstruation came to an end. One participant noted, “*When I reached menopause*, *I was relieved! Menopause brought relief from the menstrual pain and cramps I had to deal with every single month!*” (P19, age range 50–54, bachelor’s, married, full-time job). Notably, despite experiencing mild-to-moderate vasomotor symptoms during menopause, some women continued to view it as a relief from menstruation. It was therefore viewed as a time of liberation and freedom, as well as feelings of increased cleanliness and hygiene. For example, one woman stated, *“I think*, *owing to our culture and upbringing*, *we have learned that menopause is a freedom from the menstrual cycle. Therefore*, *the biggest feeling associated with menopause was that I was finally done!”* (P18, age range 55–59, bachelor’s, married, part-time job).

In the same vein, participants from post-menopausal women commented that menopause was a favourable transition, allowing them to participate in religious practices that had been restricted during menstruation: “*Now that I can fast and pray whenever I want. I feel like one chapter has closed*, *and a new chapter has opened as I see the blessings. At this time*, *a woman should take care of herself like never before”* (P6, age range 60–64, secondary education, married, housewife). Hence, the ability to observe fasting and engage in prayer without restrictions was seen as a significant shift, marking the beginning of a fresh stage characterised by spiritual benefits.

#### Theme (2): Experiencing biopsychosocial changes

This theme explores the physical and psychological symptoms experienced by perimenopausal women, their impact on daily life and the influence of societal expectations and stigma on women’s well-being, relationships and self-perceptions.

##### Menopausal symptoms: inconvenient body changes

Women’s narratives indicated that the menopausal transition is commonly seen as an *‘inconvenient change’*, covering a range of physiological, psychological, sexual, and social changes that can range in severity from mild to severe.

Hot flashes, night sweats, disturbed sleep patterns, and fatigue were commonly mentioned by most participants, at a moderate level of severity: *“I experience hot flushes randomly throughout the day and night. It feels like my body suddenly heats up and then goes away after a while. It can happen anytime*, *whether I’m sleeping or awake. I also struggle with insomnia. Sometimes*, *I also feel depressed and fatigued*, *lacking the motivation to be active or socialise.”* (P17, age range 60–64, Bachelor’s, divorced, retiring soon). Poor sleep was considered a particularly important symptom: *“My sleep quality isn’t good. I frequently wake up in the middle of the night. I mean*, *literally*, *I cannot sleep more than 2 or 1.5 hours straight.”* (P3, age range 50–54, bachelor’s, married, housewife).

Others also highlighted the impact of unforeseeable hot flashes on reducing their engagement in social activities. In particular, some women expressed discomfort and embarrassment as a consequence of excessive sweating and redness in their facial and neck areas. Issues also arose when applying makeup and wearing tight-fitting clothing, leading to social anxiety: *“Oh God*, *the hot flushes!! It can occur at any time and place. I hate it when I have to go to a social gathering and get the hot flushes*, *I get anxious*, *as I have to ask the hosts to turn on the air conditioning because all of a sudden*, *I feel hot and burning. In addition*, *the worse part that I cannot leave my hair down for a long time it gets sweaty*, *I also have become so picky with the fabrics I choose for my clothes*, *they should be 100% cotton*, *I cannot wear a bra anymore”* (P11, aged 55–59, Bachelor’s, married, retired).

Other reported physical and psychological changes included loss of body strength, joint pain, difficulty losing weight, headaches, abdominal pain, breast tenderness, mood swings, irritability, anxiety, and depression. For example, one woman asserted, *“Oh God*, *I think part of my low mood is due to perimenopause. I mean*, *during the last two years*, *I was truly depressed. I have also started experiencing severe cramps in my abdomen and breasts […] I also struggle with pain in my knees*, *perhaps as a result of my weak body and low bone density.”* (P5, aged 50–54, Bachelor’s, married, housewife).

When sexual and urinary changes were prompted, some participants noticed differences during and after menopause, such as vaginal dryness, decreased libido, discomfort during sexual intercourse, and reduced sexual activity.

Notably, women who had survived breast and cervical cancer reported experiencing more severe symptoms in comparison to women undergoing natural menopause. One participant highlighted an unexpected increase in body weight and severe vasomotor symptoms, *“Following hormonal cancer treatment*, *I suddenly experienced perimenopausal symptoms. My weight has gained unexpectedly. I also experienced severe hot flashes*, *especially at night*, *which have persisted for four years”* (P12, aged 40–44, Master’s, married, housewife). Another woman expressed feeling severe pain and discomfort during sex: *“During menopause and with the chemotherapy*, *I had severe pain and cramps in my abdominal and uterus areas. Since then*, *as a woman*, *feelings during intercourse have changed dramatically; I mean*, *the sense of comfort and pleasure during sex isn’t the same […] I believe this because*, *in my case*, *I lost my period because cancer treatment was not natural at the right time.”* (P1, aged 45–49, secondary education, married, housewife).

Only three of the sampled women reported experiencing no or very mild symptoms. One of them stated, *“I am grateful that I have not experienced any troublesome symptoms. I have heard from other women that they have had to deal with severe mood swings and depression.”* (P21, age range 55–59, PhD, married, full-time job).

##### Sociocultural expectations and influences

In addition to physical changes, menopause raised sociocultural issues that had an emotional impact on women’s mental and social well-being. This was a subject of conversation raised by many participants. For example, menopause was frequently perceived as a taboo topic, and some participants voiced difficulties in discussing menopausal symptoms with family members, including mothers, daughters, and partners. Instead, they found it more comfortable to discreetly seek information online: *“Perhaps I had felt ashamed or embarrassed to talk about it. The first time I experienced hot flashes*, *I was freaked out and hesitant to ask my daughter*, *who is studying medicine”* Consequently, she used online searches to look up information about her symptoms: *“I asked Mr. Google and looked up ‘body heat’. I read up on the topic until I realised that it was related to the disruption of my menstrual cycle. It became clear to me then that experiencing hot flashes was a normal thing for women between the ages of 45 and 50.”* (P25, age range 55–59, Bachelor’s, married, housewife).

Many married women, in particular, reported feeling hesitant to discuss menopausal symptoms with their husbands, fearing that it would make them appear older or less attractive: *“I will never tell my husband about menopause! That’s impossible… it is like if I offered him evidence that I am officially becoming old fashioned.”* (P26, aged 40–44, Master’s, married, full-time job). Hence, they opted to keep their struggles to themselves: *“I was incredibly stressed and felt disconnected from myself*, *my husband and the social world when I first experienced hot flushes. The entire experience was mentally draining*, *and I needed time to adjust to this new normal before sharing this with anyone”* (P19, aged 50–54, Bachelor’s, married, full-time job). Similarly, none of the participants had initiated conversations about sexual changes with their partners.

Another woman even expressed similar concerns using negative terms such as “*expired*” to articulate her worries:*“As women*, *want to feel desired and confident that we are still attractive*, *especially in the eyes of our partners [….] and how that would affect my husband’s perception of me as if I have become expired.”* However, the participant then countered these concerns with positive self-talk, acknowledging the natural process of aging and finding confidence in facing this journey with her partner *“But*, *I eventually realised that physical changes during menopause are a natural part of life*, *and I began to speak more positively to myself. After all*, *my husband is also getting older*, *and we are on this journey together!”* (P3, aged 50–54, Bachelor’s, married, housewife).

Though, a number of participants mentioned that they have picked up on menopausal symptoms and hot flushes from their friends, suggesting that some women may find it easier discuss menopause with friends rather than with family: *“My period stopped early when I was in my forties*, *and now*, *at 56*, *I still haven’t noticed any changes. Though some of my friends have complained about hot flashes and night sweats*, *but I haven’t had any of that.”* (P18, age range 55–59, Bachelor’s, married, part-time job).

#### Theme 3: Adjusting to the transition to menopause

This theme explores how women adapted to menopause, through self-care practices and lifestyle adjustments.

##### Placing greater priority on self

The emphasis on self-care practices emerged as a prevailing pattern among the participants. In the midst of the menopausal transition, some participants reported feeling empowered to assert control over their health and engage in significant and pleasurable activities, such as physical activity or quality time alone or with others: *“Exercise for me is like a form of self-care”* (P26, age range 40–44, Master’s, married, full-time job). *“Now that I have time to myself*, *I get to spend more quality time with myself and my husband*, *engaging in meaningful conversations*, *and taking care of myself with a full skincare routine.”* (P3, age range 50–54, Bachelor’s, married, housewife).

A decrease in family responsibilities provided women with more opportunities to prioritise their own needs and interests: “*I feel liberated and free. It seems that I have moved on from the phase of childbearing*, *and now I have ample time for self-indulgence*” (P23, aged 40–44, bachelor’s, divorced, full-time job).

Employed participants in particular viewed it as a chance to relish the fruits of their labour and move towards retirement, “*I am approaching retirement. I foresee it as a rewarding phase and a fulfilling chapter*, *InshaAllah*, *it is about allocating time for myself so I can dedicate more time to engaging in activities that bring me joy*” (P21, aged 55–59, PhD, married, full-time job).

##### Self-care techniques for symptom management

When discussing the management of symptoms, the majority of participants showed a preference for employing self-care techniques and lifestyle adjustments as a primary approach to easing menopausal symptoms. These strategies included maintaining a balanced diet, taking supplements, engaging in regular exercise, practicing meditation, prioritising sleep, and focusing on mental well-being as part of an overall holistic health approach: “*I believe by taking care of my diet*, *physical activity*, *and mental health*, *I should be able to handle the perimenopause symptoms.*” (P12, aged 40–44, Master’s, married, housewife).

Some participants used online platforms, such as YouTube channels, to look up for exercise techniques from the comfort of their home: *“I use a YouTube channel of an American middle-aged PT; she is a life coach. She’s incredible; all I do is connect my phone to the TV and do exercises in my living room…*“(P12, aged 40–44, Master’s, married, housewife).

Others reported taking soy supplements as a method for managing hot flushes: “*I took soy supplements; my gynaecologist has prescribed it for me. I took them for about 2–3 months*, *and I have noticed some improvement in my symptoms. I struggled with the hot flushes*, *but they became less severe. I stopped taking the supplement once I felt better”* (P29, age range 55–59, Bachelor’s, married, retired). Another participant considered taking herbal remedies and vitamins to help raise her estrogen hormone levels as she claimed: “*I believe that my food may impact my hot flushes and other symptoms […] but I am considering taking herbal tea and vitamins such as Omega 3 and soy pills. I previously took these supplements for a period of time to help raise my estrogen hormone levels*.” (P12, age range 40–44, Master’s, married, housewife).

Furthermore, many participants developed personalised tactics to cope with the troublesome symptoms of hot flashes and offered helpful advice for adaptation, including using a facial fan, wearing loose garments (gowns), and steering clear of hot surroundings. One woman stated, “*When I go out*, *I always bring my portable fan to cool my face down because hot flushes can suddenly hit me at any time. Additionally*, *at night*, *I make sure to wear loose and lightweight fabrics and no bra*.” (P27, aged 50–54, secondary education, married, housewife).

Nonetheless, women seemed to acknowledge the individuality of responses to self-management techniques, adopting a pragmatic perspective by recognising that successful menopause management commences with acknowledging and embracing the transformations associated with this phase of life. One participant remarked, “*I think I have come to terms with being menopausal. The key to effectively managing this phase is making peace with the idea of being menopausal*” (P12, aged 40–44, Master’s, married, housewife).

##### Devaluing medical care for menopause

The majority of participants viewed menopause as a normal physiological transition rather than a medical condition requiring professional intervention: “*I imagine that most women share my perspective. I did not feel the need to consult a medical professional for menopause*, *as I see it as a natural part of the ageing process that all women experience*” (P19, aged 50–54, bachelor’s, married, full-time job).

Another possible explanation for undervaluing medical support is that some women may underestimate its importance because menopause is often considered a socially taboo topic. They might assume it does not require medical support because other women conceal their experience with it: “*This stage of life is normal*, *I guess. All our mothers and ancestors have gone through it without the need for medical help. Therefore*, *I do not have any worries or feel stressed about it. For example*, *I saw my mum and my aunties*, *and I never noticed any changes since they lost their periods during perimenopause*.” (P9, aged 40–44, PhD, married, full-time job).

Throughout the discussion surrounding HRT, most participants demonstrated strong resistance to the utilisation of HRT owing to concerns regarding the potential heightened risk of breast and cervical cancer: “*I am against hormonal medications that would replace the oestrogen in my body. I do not want to mess with my body system*,” Instead, she articulated that “*I believe if a woman maintains a healthy diet and regular exercise; that would be enough*” (P29, aged 55–59, Bachelor’s, married, retired).

##### Practical barriers to accessing healthcare

A few participants reported facing practical barriers to accessing the national healthcare system in Saudi Arabia for menopause, including long wait times for appointments: “*It is usually tough to schedule an appointment with a doctor*.” (P11, aged 55–59, bachelor’s, married, retired).

Some also felt uneasy or stigmatised when visiting a gynecologist, especially when accompanied by their male partners or in seeking support for intimate problems: “*I found it challenging to seek medical support owing to vaginal dryness. It is challenging to communicate with my family*, *including my spouse and my daughter*, *about the necessity of consulting a gynecologist*, *as they are likely to inquire about the reasons behind it. I feel embarrassed that they might come with me to the doctor*, *and I would not want to explain the details in front of them. It is difficult for me to go to the clinic alone too*, *so I cannot get medical care without anyone knowing about it*.” (P25, aged 55–59, bachelor’s, married, housewife).

##### Proactive in medical consultations

While most participants did not seek medical advice for menopausal concerns, those who proactively discussed it with gynecologists primarily received reassurance about menopause being a natural phase in a woman’s life.

Some participants commented that medical consultations commonly emphasised regular checks, such as annual cervical and mammogram screenings, and advised Kegel exercises for urinary incontinence. However, they were less likely recommending HRT as a viable approach for managing vasomotor symptoms: *“When I saw the gynecologist*, *she did not discuss anything about hot flashes and hormonal treatments. Nonetheless*, *I brought up urinary incontinence*, *particularly triggered by laughter*, *and she suggested Kegel exercises. She also recommended pelvic floor muscle exercises and mentioned cervical screening (smear test). There was no mention of HRT advice or medications for other symptoms*, *but I have heard from my friends about a hormone that can help alleviate hot flashes.” (*P25, aged 55–59, Bachelor’s, married, housewife). Such a scenario could signify an increased level of curiosity regarding HRT treatment for menopausal symptoms and voicing dissatisfaction in the absence of explicit guidance and medical advice from healthcare professionals among some participants. Importantly, none of the participants had actively consulted with a primary care physician or a specialist in family medicine concerning issues related to menopause.

## Discussion

### Principal findings

This study is one of the few that has looked into the perceptions and experiences of women going through menopause in Saudi Arabia. Our research revealed that participants were familiar with the term ‘menopause’ and commonly viewed it as a natural transition marked by the cessation of menstruation. Most women in our study had mixed feelings about menopause, experiencing uncertainty, loss of fertility, and femininity, while also feeling relief and a sense of liberation from menstruation. Negative experiences were more prevalent among women who experienced sudden or treatment-induced menopause, as well as among those who had not given birth.

This finding is consistent with those of previous studies in other countries suggesting that women perceive menopause positively, connecting it with the cessation of menstrual discomfort and a reduced risk of pregnancy [38–42]. Furthermore, a previous study conducted in Saudi Arabia revealed that women encounter unfavourable situations during menarche, which could impact their attitudes toward menstruation [43]; hence, menopause could be seen as a form of relief.

Interestingly, certain women in our research perceived the cessation of menstruation positively from the perspective of hygiene and cleanliness, and others highlighted the possibility of menopause facilitating increased participation in religious practices. This finding aligns with prior studies carried out on Muslim women, who viewed menopause as a chance to prioritise religious duties that might be limited during menstruation [20, 44]. In Islamic traditions, women are prohibited from participating in religious activities during the menstrual period and are required to perform a ritual bath after the end of the cycle and before commencing religious activities [45].

In our study, many participants faced challenges related to cultural norms and social stigma surrounding menopause. This led to hesitance in discussing menopausal symptoms, particularly with their male partners, due to worries about how menopause might affect their relationships and how they would be perceived by others. Previous studies have highlighted similar concerns, particularly within Asian cultures, where husbands’ opinions hold significant importance alongside emphasis on the biological role of women and femininity [45]. This also prompted women in our study to discretely search for menopause information online. A similar pattern emerged in a meta-analysis of Asian women across different communities, indicating a pressing need for more reliable online health resources [46]. Additionally, consistent with previous research [47–50], our participants reported feelings of isolation and disconnection during perimenopause, suggesting inadequate social support available.

In Saudi Arabia, culture is influenced by a focus on collective and strong emphasis on family connections [51, 52]. Women’s societal roles value and prioritise family obligations and social interactions [51]. However, our research reveals that as women approach menopausal age, family and childcare responsibilities typically diminish, allowing them to place greater emphasis on their own health and well-being. The women in this study reported managing their menopausal symptoms mainly through self-care and lifestyle adjustment strategies, including nutritious diet, exercise, meditation, and self-reassurance, as well as the use of natural remedies and supplements, which is consistent with prior studies in which women opted for nonpharmacological methods to manage menopausal symptoms [20, 53, 54]. This favourable self-care approach can stem from women’s perception of menopause as a natural transition phase, representing a broader health promotion opportunity as women seek positive self-care information.

Our findings indicated that most women considered medical intervention for menopause to be unnecessary. Participants showed little interest in using HRT to ease their menopausal symptoms. While some participants had basic awareness of HRT, they opted not to pursue it, primarily due to concerns about the perceived risk of developing cancer. This reluctance may be attributed to the prevailing belief that menopause is a natural process, coupled with limited knowledge of menopausal symptoms and available treatment options. A previous study in Saudi Arabia found minimal interaction with the healthcare system in managing menopausal symptoms, underscoring the necessity for improved access to patient education and enhanced communication about menopause [55].

Our research also suggested that women in our study appeared to be less proactive in seeking medical support for menopause. Our findings revealed that none of the participants had ever discussed menopause with their general practitioners. Further, when seeking gynecologists, the consultations were mostly addressing vaginal dryness and urinary incontinence. Smear tests, pelvic floor exercises, and over-the-counter gels were usually prescribed. Women in our study also mentioned that their doctors provided reassurance, but none of them received advice on HRT treatments or other methods to prevent osteoporosis and heart disease, conditions associated with hormonal changes and increased risk in post-menopausal women. This observation is consistent with previous research conducted by Huang and colleagues, which revealed that 46% of Asian women had not received any information regarding HRT for menopausal symptoms [56, 57]. A recent survey in Saudi Arabia identified common challenges for physicians regarding HRT, including inadequate resources, limited treatment options, and patient preferences against HRT [58].

### Strengths and limitations

To our knowledge, this is the first comprehensive in-depth qualitative study of the lived experiences and perceptions of menopause in middle-aged women in Saudi Arabia. Previous menopause research conducted in Saudi Arabia has been primarily quantitative and based on descriptive cross-sectional studies [12, 14, 24–29], whilst qualitative research is less common in Saudi Arabia [59]. The interviews were conducted in Arabic via local dialects to capture the nuances and expressions of the participants, providing a deep understanding of Saudi women’s perceptions and experiences related to menopause. Our participants were purposively sampled and represented a diverse range of socioeconomic backgrounds (e.g., marital status, education, and employment). Another key strength of our study is the thoughtful design of the poster, targeting middle-aged women without overtly referencing menopausal symptoms such as hot flashes and night sweats. Informed by feedback from our PPI members, this decision addressed potential cultural sensitivities that might discourage participation and further minimised selection bias by reducing the appeal to women with more severe symptoms. Consequently, we captured a diverse range of experiences, providing a richer understanding of the needs and perspectives of Saudi women in this demographic. The choice to conduct online-based interviews may have restricted the participation of women without digital skills. However, our decision to conduct online interviews, which was based on feedback from PPI, was made to create a private space for discussing menopause comfortably and to minimise social desirability bias. This approach also allowed us to include women from various regions in Saudi Arabia, reflecting diverse local traditions, values, and socioeconomic backgrounds. We attempted to implement several strategies to improve the online interviewing process and promote engagement. However, many participants had bachelor’s degrees or higher, which limits the transferability of the findings to the wider Saudi female population, as there may be greater barriers in awareness and ability to access menopause-related information, in less-educated groups. The findings may however be somewhat transferable to other settings in the Middle East and North Africa (MENA) region where they share similar language, religious values, social norms and traditions.

### Implications for policy and practice

Previous research from Iran and Turkey suggested health education interventions are a viable approach for enhancing women’s attitudes and abilities to manage menopausal symptoms and improve quality of life [60, 61]. Similarly to previous research in Iranian women [15], our research found that women’s attitudes shifted during menopause from an uncertain and pessimistic outlook to a more optimistic and accepting one. As such, it is essential to address women’s concerns individually, taking into account their unique needs and experiences. It is also important to highlight the role of family, particularly male partners, in providing necessary empathy and support to women experiencing menopause. Many participants turned to online resources as a first step to discreetly and conveniently obtain information about menopausal symptoms and management. Thus, it is essential to enhance and establish trustworthy Arabic health information online to ensure the accuracy and reliability of such resources. Additionally, efforts should be made to address the digital divide and ensure equal access to online resources. Similarly, sharing personal anecdotes from women who have undergone similar experiences could help alleviate confusion and foster a positive outlook on menopause [62]. Establishing online support groups could also encourage open discussions, build a sense of community and inclusivity, and offer a supportive atmosphere. This, in turn, may help women navigate this transitional phase more effectively [19, 63].

Our qualitative investigation supports a ‘biocultural’ approach to understanding menopause, emphasising the complex interplay of biological, sociocultural, and environmental factors. Our findings align with those of previous studies that highlighted the limitations of the biomedical model in framing menopause as a medical issue [64–66], which may overlook the positive dimensions of menopause [42]. We advocate for a holistic approach, inclusive of self-care practices, to shift focus from troublesome symptoms to positive aspects of menopause and the opportunity to optimise health through a preventative approach, which can involve maintaining a balanced diet, engaging in regular physical activity, and prioritizing mental well-being. This aligns with our findings of a preference for using self-management strategies to cope with menopausal symptoms, which is consistent with previous research [45, 67]. Participants particularly viewed exercise as a valuable self-care practice during the menopausal transition. This highlights an opportunity to develop health behaviour promotion interventions to encourage a healthy lifestyle among middle-aged Saudi women, not only for managing menopausal symptoms but also for promoting healthy ageing and managing chronic diseases.

Our research also highlights the need for improved knowledge about HRT options and better interactions with healthcare providers for managing symptoms. Healthcare professionals need to proactively ask women about menopausal symptoms, provide education and offer a holistic approach to treatment as needed. More focus on menopause in primary care (e.g. dedicated clinics) may also be beneficial. It is also important for women to to take an active approach in seeking medical consultations to address menopausal issues.

## Conclusions

Saudi women consider menopause as a natural phase marking the end of a woman’s reproductive years. They see relief from menstrual pain, increased feelings of cleanliness, and the ability to participate in religious activities at any time of the month as positive aspects of menopause. However, they also view bothersome symptoms like hot flashes, night sweats, and feeling less attractive to significant others as negative. Self-care practices were favoured in addressing these changes, which represents an opportunity for health promotion interventions, whereas little value was placed upon medical support and HRT. The findings suggest the need to raise awareness about menopause in Saudi society using a biosociocultural approach. This could involve implementing improved online information and support, community-based educational initiatives, and establishing menopause clinics in primary care settings. Further research is needed to extend these findings by gathering insights from other key stakeholders, including healthcare providers and policymakers.

## Electronic supplementary material

Below is the link to the electronic supplementary material.

Additional file 1: Interview guide

## Data Availability

The datasets used and analysed during the current study are available from the corresponding author upon reasonable request.

## Abbreviations

HRT: Hormone replacement therapy
MENA: Middle Easter and North Africa
PPI: Public and Patient Involvement
WHO: World Health Organisation
