# Supplementary material for: Menopause knowledge, attitudes and experiences of women in Saudi Arabia: a qualitative study
Source: BMC Womens Health. 2024 Nov 25;24:624. doi: 10.1186/s12905-024-03456-7 (PMC11587664; doi:10.1186/s12905-024-03456-7)
Supplement: Supplementary file 1 — Additional file 1: Interview guide [file 12905_2024_3456_MOESM1_ESM.pdf]

# Topic Guide

WOMEN'S VIEWS AND EXPERIENCES WITH MENOPAUSE

Can I ask you how has menopause been with you so far?
[Tell a story, set a context…]

| | |
|---|---|
| Probes | • When did it start?<br>• What symptoms have you experienced?<br>• How do you see menopause?<br>• Can you tell me more about your feelings when you first noticed menopausal symptoms? |
| Prompts | - Identify common menopausal symptoms (Physical/ psychosocial / vasomotor symptoms)<br>- Positive vs negative view of menopause<br><br>*** Prompt** "To make it clearer* ***menopausal symptoms*** *can refer to any symptoms such as hot flushes, night sweats, sleep disturbance, fatigue, join aches, anxiety, depression."* |

What impact menopause has on your daily life?

| | |
|---|---|
| Probes | • What does it like to be a middle-aged woman undergoing menopausal symptoms? (self-perception, social norms, social stigma)<br>• How bothersome are menopausal symptoms? how do these affect your day-to-day life? |
| Prompts | - Interaction of menopausal symptoms (impacts on work, caring, relationship, socializing, appearance).<br>- Stereotype (social stigma / cultural factors)<br>- Perception on seeking medical consultation, family/social support etc.<br>- Preference for symptom management |

How have you been managing your symptoms?

| | |
|---|---|
| Probes | • What type of help and support you have considered/ would consider for menopausal symptoms?<br>• How do you think you would manage the symptoms?<br>• How did you perceive medical support for menopause?<br>• How do you deal with these? HRT? Is there any further support you might like?<br>• What changes you want to see regarding menopausal symptoms? |
| Prompts | - Interaction of menopausal symptoms (impacts on work, caring, socializing, appearance).<br>- Stereotype (social stigma / cultural factors)<br>- Perception on seeking medical consultation, family/social support etc.<br>- Preference for symptom management |
